# I can see (myself) clearly now: Exploring the mediating role of self-concept clarity in the association between self-compassion and indicators of well-being

**DOI:** 10.1371/journal.pone.0286992

**Published:** 2023-06-30

**Authors:** Jacob J. Coutts, Rosemary L. Al-Kire, Daniel J. Weidler

**Affiliations:** 1 Department of Psychology, The Ohio State University, Columbus, Ohio, United States of America; 2 Department of Psychological Sciences, Northern Arizona University, Flagstaff, Arizona, United States of America; University of Sharjah College of Health Sciences, UNITED ARAB EMIRATES

## Abstract

Is there a connection between loving oneself, knowing oneself, and mental well-being? Self-compassion—a construct that consists of self-kindness, acknowledgment of common humanity, and mindfulness—is associated with numerous positive outcomes including indicators of mental well-being. However, little research exists exploring the mechanism(s) by which self-compassion operates to influence these outcomes. It is possible that self-concept clarity, or the extent to which one’s self-beliefs are clearly defined and stable, acts as such a mechanism. In the current study, we explored the mediating role of self-concept clarity in the associations between self-compassion and three indicators of mental well-being: perceived stress, depressive symptomatology, and life satisfaction. Self-compassion was significantly associated with each of the three indicators of well-being. Additionally, self-concept clarity statistically mediated the relationships between self-compassion and depressive symptomatology, perceived stress, and satisfaction with life. The results of this study suggest a potential mechanism by which self-compassion is associated with greater well-being.

## Introduction

Given the rapid rise in depression and associated outcomes, psychologists have increasingly sought ways to treat depressive symptomatology and improve quality of life and mental well-being [1, 2]. One well-documented way to enhance well-being is the practice of self-compassion [1, 3]. Although there is a large body of literature suggesting self-compassion improves well-being, research on mechanisms explaining *how* self-compassion enhances well-being is sparse. This understanding is crucial for practitioners and therapists looking to integrate self-compassion into their practice as well as researchers seeking to map the nomological network of self-compassion. In this study, we contribute to this literature by exploring self-concept clarity as a potential mediator in the association between self-compassion and mental well-being. Additionally, we test this association across three common indicators of well-being (i.e., depressive symptomatology, perceived stress, and satisfaction with life) to determine whether this effect is robust across different conceptualizations of mental health or only related to specific indices of mental well-being.

### Self-compassion

Self-compassion is derived from concepts in Buddhist philosophy and consists of three components: 1) self-kindness, 2) common humanity, and 3) mindfulness. Self-kindness is the act of responding to pain or disappointment by being caring and considerate to the vulnerable self. Common humanity involves one making an active effort to view their negative experiences as part of the larger human experience—that is, these experiences are universal and not isolated incidents that only afflict oneself [4]. Mindfulness involves equanimity and carefully considering and accepting one’s painful thoughts/feelings without over-identifying with them or suppressing them [4].

Many studies have documented the benefits of self-compassion. For example, self-compassion is associated with outcomes like healthy coping mechanisms, lower scores on depression inventories, lower levels of perceived stress, higher levels of self-reported happiness, and greater academic achievement [5, 6]. Despite this body of work, few researchers have explored the mechanisms by which these effects occur. Exploring the processes that explain these relationships (via tests of mediation) would provide a more complete understanding of self-compassion as a psychological construct and the ways in which it is associated with well-being outcomes.

### Indicators of mental well-being

Mental well-being can be broadly conceptualized as a global composite of various psychological outcomes and is optimized when an individual has greater positive affect (e.g., optimism) and less negative affect (e.g., depression) [7, 8]. Herein, we focus our research on three important indicators of mental well-being: depressive symptomatology, perceived stress, and satisfaction with life. Although not fully comprehensive, these aspects were chosen as they represent well-being related constructs that are central indicators of psychological functioning [9–11]. Additionally, these components capture distinct facets of well-being, reflective of both positive (i.e., satisfaction with life) and negative (i.e., depression and stress) factors.

#### Depressive symptomatology

Depression is a prevalent psychological disorder that is associated with adverse psychological outcomes such as anxiety, feelings of guilt and worthlessness, low self-esteem, and neuroticism [12]. Depression can be alleviated through biological treatments, therapy, or alternative practices such as mindfulness [13, 14]. Another way to alleviate symptoms of depression may be to target self-compassion. Previous work suggests that the active components of self-compassion (i.e., self-reflection, self-forgiveness, and mindfulness) alleviate negative cognitions associated with depression [15]. Accordingly, depressive symptomatology is inversely associated with self-compassion [1, 6].

#### Perceived stress

Perceived stress is associated with anxiety and depression and can, among other things, negatively affect one’s mental health, memory formation, and satisfaction with life [16, 17]. Individuals may experience stress from many sources including academic settings and jobs or careers [18]. Self-compassionate individuals tend to experience less anxiety and more effectively cope with negative life events [16]. Increases in self-compassion may lead to a decrease in stress and increase in happiness and life satisfaction [4].

#### Satisfaction with life

Life satisfaction is the result of a cognitive and judgmental process where one compares their current situations to an ideal standard [19]. Satisfaction with life correlates with other indicators of well-being (e.g., subjective happiness and optimism) and has been identified as a protective factor against nonsuicidal self-injury, suicidal ideation, and suicide attempts [20–22]. Moreover, practicing self-compassion can increase healthy outcomes like satisfaction with life [5]. Individuals with a greater self-concept clarity tend to report higher levels of satisfaction with life [23].

### Self-concept clarity

Self-concept clarity is the extent to which beliefs about the self are internally consistent, confidently defined, and stable over time [24]. Self-concept clarity is associated with a variety of well-being indices including higher self-esteem, lower levels of perceived stress, grit, and positive identity formation [23–27]. Additionally, self-concept clarity is a predictor of three important indicators of mental well-being: perceived stress [23, 28], depressive symptomatology [28, 29], and satisfaction with life [30].

#### Self-concept clarity as a mediator between self-compassion and well-being

Given its underlying importance in the structure and function of the self, self-concept clarity has been proposed as a mediator in models of psychological processes and well-being [23, 30], but has not yet been examined for self-compassion. Theoretical conceptualizations of self-concept clarity would suggest that greater self-concept clarity should emerge from practicing self-compassion. Self-compassion involves taking the time to kindly and intentionally reflect on who one is and their strengths and weaknesses [4, 16]. Through diligent, equanimous reflection of themselves, individuals should have a clearer, more stable sense of who they are (i.e., greater self-concept clarity) than someone who does not self-reflect in this way; especially regarding their weaknesses [31]. As self-concept clarity is fundamental to well-being related outcomes, this clearer sense of self, engendered through self-compassionate cognition and behavior, could in turn lead to enhanced well-being.

There is empirical precedent to believe self-concept clarity plays a mechanistic role between self-compassion and well-being. Specific processes involved in self-compassion promote greater clarity of the self. Hanley and Garland [32] found self-concept clarity mediated the relationship between mindfulness (one of the components of self-compassion) and mental well-being. Additionally, Dummel [33] found that increased mindfulness led to greater levels of self-concept clarity, which decreased internal conflict and increased confidence in one’s attitudes. But mindfulness is just one aspect of self-compassion; common humanity and self-kindness are also important. By thinking about one’s experiences as part of a greater human experience and realizing that one is not alone in their behavior or experiences, one’s self concept and identity as a human could be strengthened. Additionally, kindness toward oneself in the wake of disappointment could lead to a clearer sense of self since one is not avoiding thinking about pain or personal weaknesses.

### Overview of the present study

The primary goal of the present research was to explore the mediating role of self-concept clarity in the relationship between self-compassion and indicators of mental well-being. More specifically, we wanted to answer the question: Does self-concept clarity statistically mediate the relationship between self-compassion and depressive symptomatology, perceived stress, and satisfaction with life? We hypothesized that self-compassion would be negatively associated with depressive symptomatology and perceived stress and positively associated with satisfaction with life, and that these three relationships would be statistically mediated by self-concept clarity.

## Method

### Ethics statement

This study was approved by the ethics board at Northern Arizona University and was in accordance with the ethical guidelines of the American Psychological Association. Written informed consent was obtained from every participant in the study and the data were anonymized prior to analysis.

### Participants and procedure

A total of 253 undergraduate students at a university in the southwestern United States (191 women, 44 men; aged 18–52 [*M*_age_ = 21.22, *SD* = 5.76]) participated in the study. The sample predominantly identified as Caucasian/White (64.7% Caucasian/White, 16.2% Hispanic/Latino[a], 8.9% Racially/Ethnically Mixed, 5.5% Asian/Asian-American/Pacific Islander, 2.1% African American/Black, 2.1% Alaskan Native/Native American, and 0.4% declined to state).

The research design was cross sectional. Participants were convenience sampled over the course of two semesters through an online participant recruitment database and data were collected via an online survey. The online survey was part of a larger study. All participants who agreed to an informed consent completed a questionnaire that concluded with a debrief form detailing the purposes of the study. The measures consisted of a demographic questionnaire followed by measures of self-compassion, depressive symptomology, satisfaction with life, perceived stress, and self-concept clarity (other constructs were present in the questionnaire such as compassion toward others, however analyses were only conducted and reported on variables pertinent to the present study). The average time to complete the questionnaire was approximately 35 minutes and participants were compensated with course credit and entered into a raffle for a number of gift cards. Participants were excluded from the analysis if they had missing data on one or more measures present in the model.

#### Sample size determination

The MCpowrMed Rshiny application was used for sample size determination by way of a power analysis. This application determines sample sizes based on Monte Carlo resampling confidence intervals. (At the time of study planning, no functions existed for sample size determination based on percentile bootstrap confidence intervals. We felt this was software a reasonable choice since percentile bootstrap and Monte Carlo confidence intervals have similar levels of power in mediation analysis and the Monte Carlo generally leads to a more conservative sample size determination [38]). Based on prior data collected in the lab, we expected a standardized regression coefficient around *β* = 0.30 for the effect of self-compassion on self-concept clarity (i.e., the *a* path), effect sizes averaging *β* = 0.20 for the effects of self-concept clarity on indicators of well-being (i.e., the *b* paths), and an average standardized direct effect (i.e., *c*′) of *β* = 0.20. With these estimates, MCpowrMed suggested a sample size of 208 to reach a power of 0.80. Data collection stopped at the end of the semester that this sample size was reached.

### Measures

The reliability of each scale was computed by McDonald’s Omega. Omega, an alternative to Cronbach’s Alpha, was obtained from OMEGA, an SPSS and SAS macro [34]. Unlike Cronbach’s Alpha, McDonald’s Omega does not assume tau equivalence, and thus many methodologists recommend calculating Omega instead of Alpha for an estimate of reliability [34].

#### Self-compassion

Participants completed the 12-item short form of the Self-Compassion Scale [35; obtained McDonald’s *ω* = .83] developed from the 26-item Self-Compassion Scale [4]. Items include statements such as, “When I fail at something important to me I become consumed by feelings of inadequacy.” Responses are indicated on a five-point scale ranging from “*Almost Never*” to “*Almost Always*.” Total scores were created by taking the mean of the items, with higher scores on the scale suggesting greater self-compassion.

#### Depression

Participants completed the 20-item CES-D [36; obtained *ω* = .93]. Items asked participants to rank how often, within the past week, they experienced symptoms such as, “I was bothered by things that usually don’t bother me.” Responses were given on a four-point scale ranging from “*Rarely or Some of the Time*” to “*Most or Almost All the Time*.” Total scores were created by summing the items, with higher scores on the scale suggesting greater depressive symptomatology.

#### Satisfaction with life

Participants completed the five-item Satisfaction with Life Scale [19; obtained *ω* = .89]. Items include statements such as, “In most ways, my life is close to my ideal.” Responses were given on a seven-point scale ranging from “*Strongly Disagree*” to “*Strongly Agree*.” Total scores were created by summing the items, with higher scores on the scale suggesting greater satisfaction with life.

#### Perceived stress

Participants completed the 10-item Perceived Stress Scale [37; obtained *ω* = .88]. Items include statements such as, “In the last month, how often have you felt that you were unable to control the important things in your life?” Responses were given on a five-point scale ranging from “*Almost Never*” to “*Almost Always*.” Total scores were created by summing the items, with higher scores on the scale suggesting greater perceived stress.

#### Self-concept clarity

Participants completed the 12-item Self-Concept Clarity Scale [24; obtained *ω* = .91]. Items include statements such as, “If I were asked to describe my personality, my description might end up being different from one day to another day.” Responses were given on a five-point scale ranging from “*Almost Never*” to “*Almost Always*.” Total scores were created by summing the items, with higher scores on the scale suggesting a higher self-concept clarity.

## Results

Analyses were conducted in PROCESS v4.0 [38] and R. A total of 10,000 bootstrap samples were used to construct percentile bootstrap confidence intervals with seed number 12122017. (If persons wish to reproduce the results, entering this as the seed will allow users to obtain the same bootstrap estimates; the data are available as S1 Data). A total of 18 participants were excluded listwise due to missing data on one or more measures of focus in the analyses. Data from 235 participants were analyzed.

### Primary analyses

The McDonald’s *ω*s, means, standard deviations, and bivariate correlations amongst all the study variables are reported in Table 1.

**Table 1:** Obtained McDonald’s omegas, means, standard deviations, and bivariate correlations amongst the study variables.

	1	2	3	4	5	6	*M* (*SD*)
1. CES-D (*ω* = .93)	-	-	-	-	-	-	20.71 (12.17)
2. PSS (*ω* = .88)	.72***	-	-	-	-	-	29.92 (7.15)
3. SC (*ω* = .83)	−.52***	−.61***	-	-	-	-	2.91 (.63)
4. SCC (*ω* = .91)	−.57***	−.58***	.50***	-	-	-	36.45 (9.99)
5. SWL (*ω* = .89)	−.57***	−.54***	.46***	.37***	-	-	23.71 (6.76)
6. Age	−.15*	−.16*	.06	.19**	.04	-	21.16 (5.58)
7. Gender	−.04	−.16*	.11	.18**	.00	−.03	-

*Note*. Abbreviations are CES-D (Center for Epidemiological Studies–Depression); PSS (Perceived Stress); SC (Self-Compassion); SCC (Self-Concept Clarity); SWL (Satisfaction with Life).

*p < .05

**p < .01

***p < .001

#### Hypothesis 1

**Fig 1:**
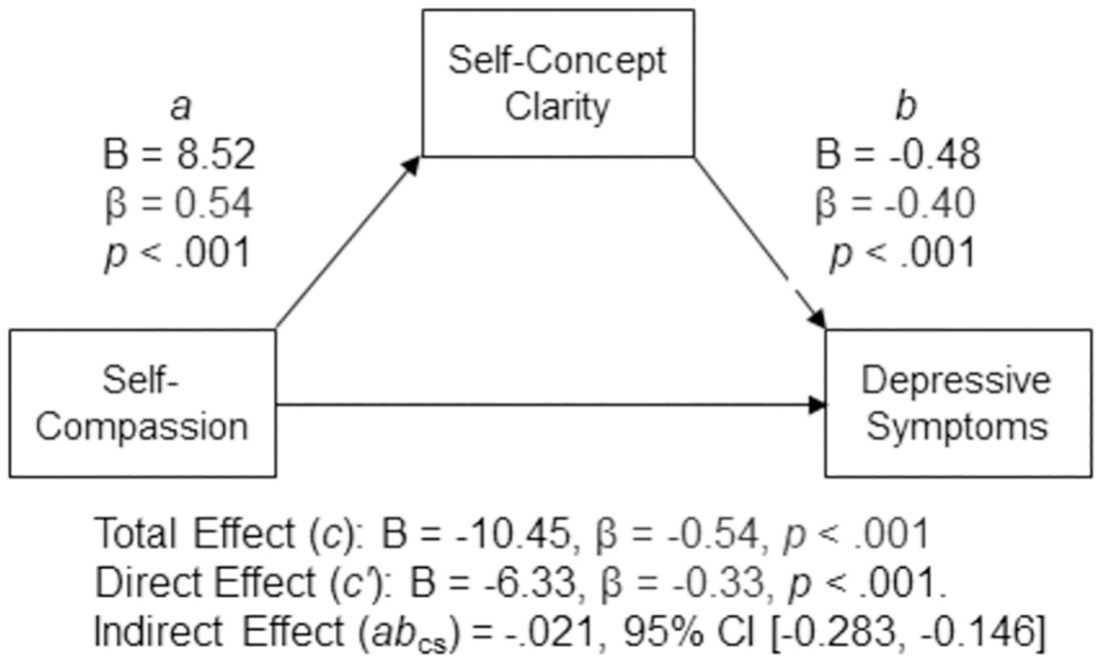
Standardized (β) and unstandardized (B) regression coefficients for the relationship between self-compassion and depressive symptoms as mediated by self-concept clarity.

Self-compassion was negatively associated with depressive symptomatology (*c* path; B = –10.45, β = –0.54, *p* < .001, 95% CI [–12.539, –8.361]). The analysis suggested that self-compassion indirectly statistically predicted depressive symptomatology through self-concept clarity. Participants who self-reported higher levels of self-compassion also reported higher levels of self-concept clarity (*a* path; B = 8.52, β = .54, *p* < .001, 95% CI [6.795, 10.242]), and participants who self-reported higher levels of self-concept clarity self-reported lower levels of depressive symptomatology after controlling for self-compassion (*b* path; B = –0.48, β = –0.40, *p* < .001, 95% CI [–.628,–.340]). The 95% percentile confidence interval for the completely standardized indirect effect (*ab*_cs_ = –0.21) did not contain zero (95% CI [–0.283, –0.146]). The direct effect was also significant, suggesting that self-compassion was negatively associated with depressive symptomatology independent of its effect through self-concept clarity (*c′* path; B = –6.33, β = –0.33, *p* < .001, 95% CI [–8.603, –4.050]). A summary of the model results can be found in Fig 1.

#### Hypothesis 2

**Fig 2:**
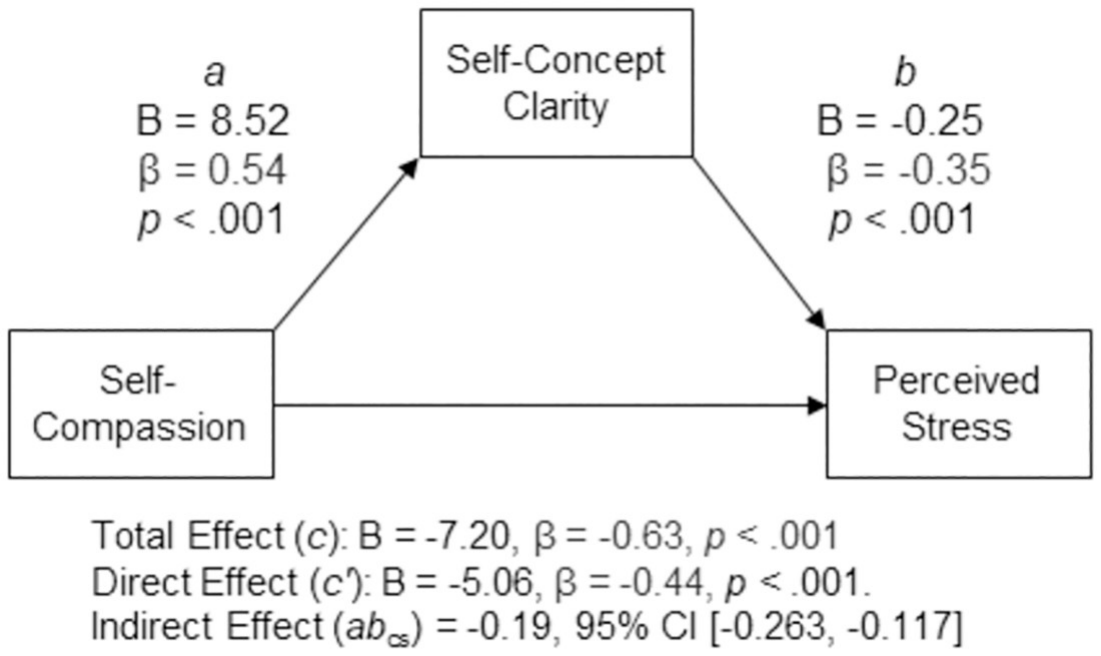
Standardized (β) and unstandardized (B) regression coefficients for the relationship between self-compassion and perceived stress as mediated by self-concept clarity.

Self-compassion was negatively associated with perceived stress, (*c* path; *B* = –7.20, β = –0.63, *p* < .001, 95% CI [–8.340, –6.070]). The analysis suggested that self-compassion indirectly statistically predicted perceived stress through self-concept clarity. Participants who self-reported higher levels of self-compassion also reported higher levels of self-concept clarity (*a* path; *B* = 8.52, β = 0.54, *p* < .001, 95% CI [6.795, 10.242]), and participants who self-reported higher levels of self-concept clarity self-reported lower levels of perceived stress after controlling for self-compassion (*b* path; *B* = –0.25, β = –0.35, *p* < .001, 95% CI [–.331,–.174]). The 95% percentile confidence interval for the completely standardized indirect effect (*ab*_cs_ = −0.19) did not contain zero (95% CI [–0.263, –0.117]). The direct effect was also significant, suggesting that self-compassion was negatively associated with perceived stress independent of its effect through self-concept clarity (*c′* path; *B* = –5.06, β = –0.44, *p* < .001, 95% CI [–6.302, –3.810]). A summary of the model results can be found in Fig 2.

#### Hypothesis 3

**Fig 3:**
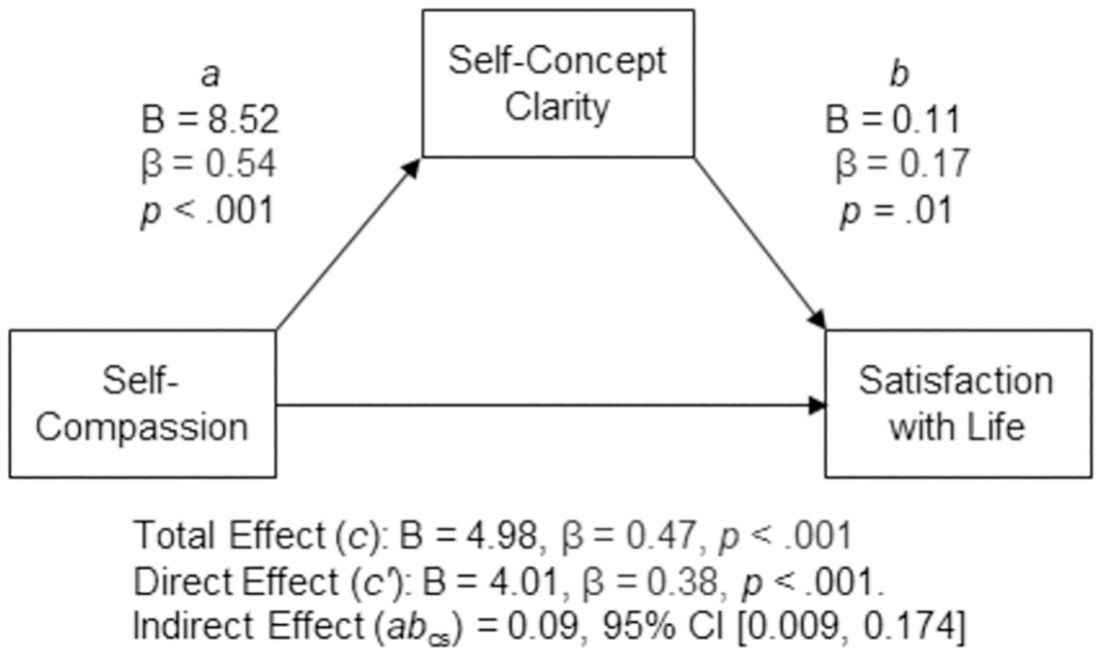
Standardized (β) and unstandardized (B) regression coefficients for the relationship between self-compassion and satisfaction with life as mediated by self-concept clarity.

Self-compassion was positively associated with satisfaction with life, (*c* path; *B* = 4.98, β = 0.47, *p* < .001, 95% CI [3.766, 6.198]). The analysis suggested that self-compassion indirectly statistically predicted satisfaction with life through self-concept clarity. Participants who self-reported higher levels of self-compassion also self-reported higher levels of self-concept clarity (*a* path; *B* = 8.52, β = 0.54, *p* < .001, 95% CI [6.795, 10.242]), and participants who self-reported higher levels of self-concept clarity self-reported higher levels of satisfaction with life after controlling for self-compassion (*b* path; B = 0.11, β = 0.17, *p* = .01, 95% CI [.024, .204]). The 95% percentile confidence interval for the completely standardized indirect effect (*ab*_cs_ = 0.09) did not contain zero (95% CI [0.009, 0.174]). The direct effect was also significant, suggesting that self-compassion was positively associated with satisfaction with life independent of its effect through self-concept clarity (*c′* path; *B* = 4.01, β = 0.38, *p* < .001, 95% CI [2.588, 5.441]). A summary of the model results can be found in Fig 3.

## Discussion/conclusions

The purpose of this study was to investigate the associations among self-compassion, self-concept clarity, and psychological well-being. We posited that self-compassion enhances well-being indirectly (at least in part) through self-concept clarity. We found that self-compassion and self-concept clarity were significantly associated with three indicators of well-being: lower depressive symptomatology and perceived stress and higher satisfaction with life. Further, we found that those who reported greater self-compassion also reported greater levels of self-concept clarity. Lastly, consistent with our hypothesized model, self-concept clarity statistically mediated the relationships between self-compassion and all three indices of well-being.

These results are consistent with prior literature demonstrating associations among these constructs individually. For instance, our findings replicate previous research on self-compassion and self-concept clarity which show strong associations across a variety of indicators of well-being [3, 16, 26]. Moreover, these findings build on and extend work that has demonstrated a mechanistic role of self-concept clarity in the association between mindfulness and well-being [32].

This study contributes to the psychological literature on self-compassion. Despite a robust literature demonstrating the efficacy of self-compassion in promoting or causing positive well-being [5, 6], to our knowledge, this study is one of the few empirical investigations which tests a potential mediator of the associations between self-compassion and well-being. This provides an important step toward building and refining theory related to self-compassion and highlights promising avenues for future research.

These results also have practical implications for practitioners and psychological health interventions. To promote greater well-being and reduce depressive symptomatology, practitioners may seek to build on their current practices by emphasizing self-concept clarity as an important outcome of the practice of self-compassion. However, these findings may also be useful outside of clinical settings as well. Self-compassion may also be efficacious in contexts such as the workplace or college environment—especially the latter, where college students have higher-than-average incidence of mental illness [39]. The self is a universal human experience that can also be emphasized when teaching anyone about mindfulness, common humanity, and self-kindness.

### Limitations and future directions

Although the results of this study support prior literature and suggest interesting patterns within the nomological network of self-compassion, the study is not without limitations. First, this was a cross-sectional study that relied on correlational data. Utilizing a longitudinal model with experimental data would allow researchers to rigorously establish cause-and-effect and reduce the limitations associated with cross-sectional designs (e.g., cohort effects). We encourage researchers (including ourselves) to continue this research experimentally and/or longitudinally to test causal relationships.

The study lacked diversity, especially related to gender and sexuality. It could be useful to sample more persons in the LGBT+ community to test whether similar patterns of associations emerge. Specifically, members of these communities (e.g., “questioning”) may struggle more with clarity and formation of self-identity [40]. LGBT+ persons may also exhibit differences in the patterns of self-compassion or self-concept clarity that would be important to investigate [41, 42]. Since prior research indicates that self-compassion and self-concept clarity can be a protective factor against suicidality among LGBT+ youth [41, 43], future studies could include gender or LGBT+ identity as a moderator in a conditional process model (i.e., a model where the indirect effect is moderated). This could potentially provide beneficial insight for ways to combat some of the stressors faced by marginalized groups and promote greater psychological well-being.

Despite providing evidence for the mechanistic role of self-concept clarity in the association between self-compassion and three indices of well-being, additional work should be devoted to defining additional mechanisms explaining this relationship. It is likely that self-concept clarity is only a piece to understanding a larger picture of the relationship between self-compassion and well-being. Researching these mechanisms is beneficial for psychological theory and practitioners trying to teach and learn self-compassion.

#### Statistical and methodological constraints

Our primary analyses consisted of simple mediation models. These models are often not complex enough to fully represent real-world processes, may suffer from biased parameter estimates due to omitted variables, and violate testing assumptions—especially in the absence of experimental and/or longitudinal designs [38, 44]. For instance, although we argued that self-concept clarity mediates the association between self-compassion and mental well-being due to the reflective and self-kindness components that encompass self-compassion, it could be argued instead that self-compassion mediates the relationship between self-concept clarity and mental well-being. These models cannot be mathematically distinguished (indeed, from several other models involving the same variables); thus, the ordering of the variables in the model are based solely on theoretical argument [45]. Although it is useful to know that these variables predict one another in a statistical sense as our results suggest, future research endeavors should collect experimental data and provide more evidence that these variables causally influence one another in the hypothesized order.

### Conclusion

This study explores constructs that have the potential to improve a person’s mental well-being (and thus their quality of life). Research on self-compassion and well-being continues to grow, and as such, it is important to understand the ways and conditions in which self-compassion can be beneficent. This improved understanding is helpful for scholars, clinicians, professionals, and laypersons alike. With increasing depression and anxiety levels, research investigating self-compassion has the power to equip persons with a learnable skill that can help ameliorate the negative impact of psychological disorders or everyday stressors.

## Supporting information

S1 Data (CSV)
